# Phenotype and target-based chemical biology investigations in cancers

**DOI:** 10.1093/nsr/nwy124

**Published:** 2018-11-01

**Authors:** Guo-Qiang Chen, Ying Xu, Shao-Ming Shen, Jian Zhang

**Affiliations:** Department of Pathophysiology, Key Laboratory of Cell Differentiation and Apoptosis of Chinese Ministry of Education, Shanghai Jiao Tong University School of Medicine (SJTU-SM), Shanghai 200025, China

**Keywords:** chemical biology, chemical compounds, target, cancer, leukemia

## Abstract

Chemical biology has been attracting a lot of attention because of the key roles of chemical methods and techniques in helping to decipher and manipulate biological systems. Although chemical biology encompasses a broad field, this review will focus on chemical biology aimed at using exogenous chemical probes to interrogate, modify and manipulate biological processes, at the cellular and organismal levels, in a highly controlled and dynamic manner. In this area, many advances have been achieved for cancer biology and therapeutics, from target identification and validation based on active anticancer compounds (forward approaches) to discoveries of anticancer molecules based on some important targets including protein-protein interaction (reverse approaches). Herein we attempt to summarize some recent progresses mainly from China through applying chemical biology approaches to explore molecular mechanisms of carcinogenesis. Additionally, we also outline several new strategies for chemistry to probe cellular activities such as proximity-dependent labeling methods for identifying protein-protein interactions, genetically encoded sensors, and light activating or repressing gene expression system.

## INTRODUCTION

Chemistry plays key roles in helping to decipher and manipulate life activities, and the interdependency of chemistry with biology, pharmacology and medicine has been shown to be of great synergistic value [1,2]. For example, discoveries of the potent chemical probes JQ1 [3] and I-BET (inhibitor for bromodomain and extra terminal) [4] are triggering revolutionary progress in our understanding of bromodomain biology and pharmacology [5,6]. Thus, a thriving interdisciplinary scientific area, chemical biology has been attracting a lot of interest [7].

**Figure 1.**
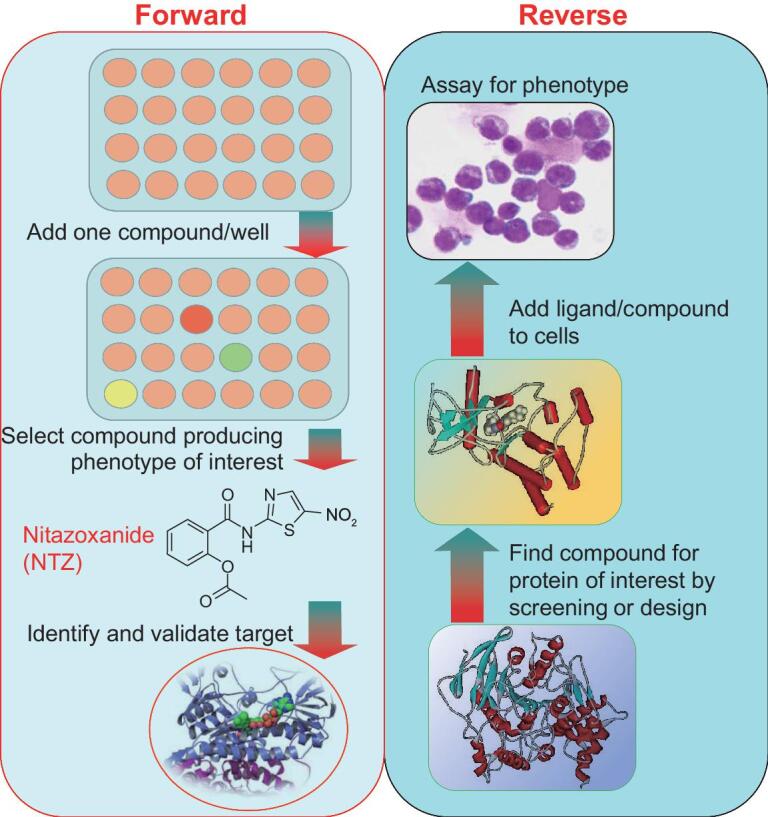
General flow chart of forward/compound-based and reverse/target-based chemical biology approaches.

Different from classical biochemistry, which focuses on the understanding of endogenous chemical processes in living systems, chemical biology employs the methods and techniques of chemistry to investigate biological phenomena. In particular, chemical biology aims to use exogenous chemical probes to interrogate, modify, and manipulate biological processes at the cellular and organismal levels in a highly controlled, reversible and dynamic manner. By analogy to classical genetics, chemical biology can also employ forward and reverse approaches (Fig. 1). Forward or phenotype-based chemical biology begins by screening compounds to trigger interesting phenotypes of cells or organisms, after which the biological target(s) of the interesting compound are identified. Reciprocally, reverse or target-based chemical biology usually starts with known targets that have been validated to play a critical role in a particular signaling pathway, biological activity or disease of interest.

The utility of chemical biology is also appreciated by scientists in China. The National Climbing Program for Basic Research initiated in the late 1980s, the precedent of the National Basic Research Program of China (973 Program), funded a project entitled ‘chemical studies in biological processes’ [8]. From then on, scientists in China made greater contributions to chemical biology research, especially forward chemical biology based on screening of active compounds, such as the application and target identification of *all-trans* retinoic acid (ATRA) and arsenic trioxide (As_2_O_3_) in the treatment of acute promyelocytic leukemia (APL), a unique subtype of acute myeloid leukemia (AML) [9,10], and the chemical induction of pluripotent stem cells from mouse somatic cells [11–13].

To push the development of chemical biology as a multidisciplinary research priority area, the National Natural Science Foundation of China (NSFC) launched a major research program on chemical biology in 2005, named ‘investigations on signal transduction processes utilizing small chemical probes’, which focused on the development of new techniques and methods to detect the information of signaling processes, exploring chemical compound-based signaling mechanisms of cellular functions, and the discovery of targets and lead compounds based on signal transduction processes [8]. Following the program, a new plan entitled ‘dynamic modification of biological macromolecules and chemical interference’ was initiated by the NSFC in 2017.

Due to extremely high morbidity and mortality worldwide, cancers are always the main focus of medical research. Over the past two decades, potential therapeutic targets for cancers have been generated by exponential growth in the amount of genomic information available, with the support of data from various kinds of other omics such as epigenomics, transcriptomics, proteomics and metabolomics, as well as synthetic lethal screens based on RNA interference, clustered regularly interspaced short palindromic repeats/Cas systems and classical hypothesis-driven approaches [14–16]. However, it is estimated that approved drugs are available for only 5% of the 500 or so cancer-causing gene products [17,18]. Available chemical modulators (inhibitors for oncogenic drivers and activators for dysfunctional tumor suppressor proteins) are applicable to less than 10% of the cancer proteome. Moreover, at least 10% of all pathogenic cancer driver genes are considered to be druggable with current technologies, but they have not yet been chemically explored. Therefore, there is an urgent medical need to extend the chemical targeting of cancers, which will be essential if we are to better understand the functioning of cancer-related genes and pathogenic networks, and develop personalized, precision therapeutic strategies for cancer patients. This review tries to highlight some, but not all examples, of recent progress, mainly from China, in the discovery of chemical tools and the application of chemical biology approaches against cancer.

## CHEMICAL LIBRARY

A chemical or drug library is a collection of stored chemicals that is usually used in high-throughput screening systems to identify chemical probes of disease-related targets and potential starting points for drug discovery. Each chemical library should have associated information collected in the form of a database, such as chemical structure, purity, quantity and known potential biological activities. A rational chemical library with wide chemical structure space will increase the chance of finding a ‘hit’ in the high-throughput screen. Considering that many active chemicals identified through high-throughput screening are not druggable, drug-like properties such as solubility and bioavailability are important to chemicals collected in a drug library.

Chemical compounds include natural and synthetic compounds. Natural compounds or products derived from plants such as traditional Chinese medicine (TCM) herbs, minerals, microorganisms and animals have been used as valuable sources of clinical drugs or chemical probes to identify new mechanisms for life activities. Although natural compounds have common disadvantages such as access and supply, processing complexities of natural product chemistry, inherent development delays and entangled interests regarding intellectual property rights, they have been attracting significant attention for the development of novel chemotherapeutics against cancers because of their remarkable efficacy and generally low toxicity. It is estimated [19] that more than half of the anticancer drugs approved by the Food and Drug Administration of American (FDA) between 1981 and 2014 originated from natural products and/or their derivatives.

Natural products alone cannot be used to build a huge library with diverse structures. The synthesis of natural products has been one of the mainstays of organic chemistry over the last century, and great progresses in the synthesis of natural product analogs has been achieved [1]. Combinatorial chemistry allows for the synthesis of vast numbers of compounds. Diversity-oriented synthesis (DOS) can provide an efficient manner to generate such a library [20]. By using the DOS library, many active molecules have been discovered that modulate protein–protein interactions (PPIs), transcription factor activity and multidrug resistance, as reviewed by He *et al*. [21]. Moreover, the synthetic approach enables the mass production of some rare natural products, as well as optimized versions of primary active compounds identified in a high-throughput screening.

A chemogenomic library is a relatively small library containing hundreds-to-thousands (rather than millions) of selective small molecules with known or potential targets or functions, of which the majority target G protein-coupled receptors, kinases and ion channels, the most common molecular targets for drug discovery [22]. Using such a library can greatly increase the opportunity to repurpose a drug that acts on a novel pathway or target [23]. It should be pointed out that dark chemical matter, those small molecules in a screening collection that have never shown biological activity despite having been exhaustively tested in high-throughput screening, has also been reported to occasionally result in potent hits with unique activity and clean safety profiles, which makes them valuable starting points for lead optimization efforts [7,24].

**Figure 2.**
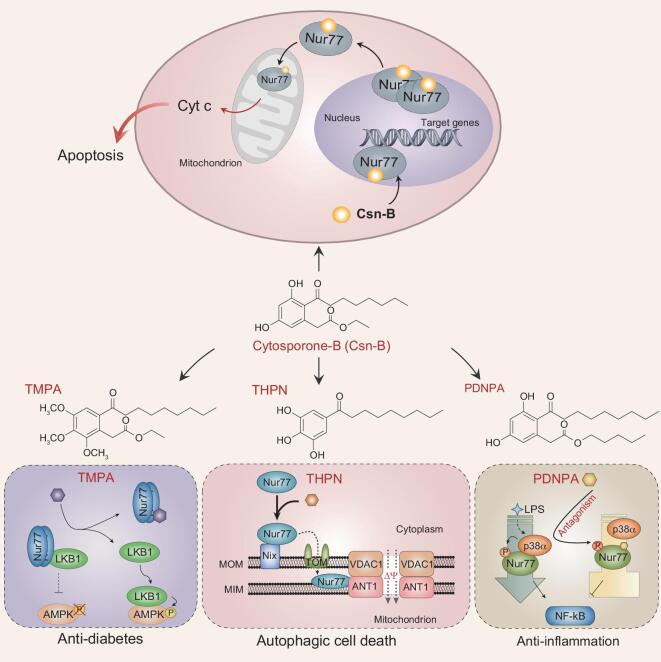
Chemical biology investigations based on Nur77-targeting compound Csn-B and its derivatives. Csn-B specifically binds to the ligand-binding domain of Nur77 and subsequently activates its transactivational activity towards target genes, including Nur77 itself. Then, the increased Nur77 is translocated to the mitochondria to cause cytochrome C (Cyt c) release, inducing apoptosis and retarded xenograft tumor growth. Three other derivatives of Csn-B, including TMPA (ethyl 2-[2,3,4-trimethoxy-6-(1-octanoyl)phenyl]acetate), THPN (1-(3,4,5-trihydroxyphenyl)nonan-1-one) and PDNPA (n-pentyl 2-[3,5-dihydroxy-2-(1-nonanoyl)phenyl]acetate), can exert different functions in decreasing glucose, inducing autophagy and inhibiting inflammation by modulating various signaling pathways.

Here, we want to introduce a small compound library from Q. Wu’s group at Xiamen University, which specifically targets to the orphan nuclear receptor Nur77 (also known as NR4A1, NGFIB, TIS1, NAK-1, TR3 or N10), a member of the nuclear receptor NR4A subfamily. Nur77 is an immediate early gene-encoded unique transcription factor that is rapidly and transiently induced in response to changes in the extracellular environment. In addition to its transcriptional function, Nur77 also presents a non-genomic signaling function through its physical interactions with various signaling proteins, such as p53, hypoxia inducible factor (HIF)-1α, protein arginine N-methyltransferase 1, protein kinase C, retinoid X receptor (RXR) and Wnt (wingless)/β-catenin signaling, thereby modulating a wide range of important biological functions, including cell cycle progression, apoptosis, autophagy, inflammation, metabolism and energy homeostasis. Nur77 expression and signaling transduction is regulated in many cancers, thus providing an important molecular target for drug screening [25]. Wu’s group identified cytosporone-B (Csn-B, Fig. 2), which is extracted from a mangrove endophytic fungus, as the first naturally occurring agonist for Nur77 [26]. Based on the structure of Csn-B, they designed and synthesized more than 300 derivatives to construct a unique library of small-molecule compounds that specifically target Nur77. Thereafter, they extensively explored the molecular mechanisms of Nur77 in the regulation of glucose metabolism, autophagy, inflammation and carcinogenesis. For example, they reported that the chemical compound TMPA (ethyl 2-[2,3,4-trimethoxy-6-(1-octanoyl)phenyl]acetate) can bind to the ligand-binding domain of Nur77, causing a Nur77 conformational change and resulting in the disruption of association of Nur77 with liver kinase B1 (LKB1), the latter having an important role in governing energy homeostasis by regulating the activity of the AMP-activated protein kinase (AMPK) energy sensor. As a result, LKB1 is released into the cytoplasm to phosphorylate and activate AMPK, finally downregulating glucose levels in diabetic mice (Fig. 2) [27]. They also demonstrated that the compound THPN (1-(3,4,5-trihydroxyphenyl)nonan-1-one) from their compound library triggers the movement of Nur77 into the mitochondria by interaction with Nix, where Nur77 is located in the mitochondrial inner membrane and interacts with ANT1, and then causes the opening of the mitochondrial permeability transition pore and the depolarization of the mitochondrial membrane, eventually leading to the irreversible autophagic death of melanoma cells. In a mouse model of spontaneous skin melanoma, they confirmed that THPN induced autophagic cell death in a Nur77-dependent manner, which further inhibits the development and metastasis of melanoma (Fig. 2) [28]. This work not only elucidated a new mechanism by which Nur77 participates in autophagic cell death induction through a mitochondrial signaling pathway, but also demonstrated that using THPN to induce autophagic death of melanoma cells can overcome the resistance of melanoma cells to drug-induced apoptosis. Additionally, the small-molecule compound PDNPA (n-Pentyl 2-[3,5-dihydroxy-2-(1-nonanoyl)phenyl]acetate) obviously inhibits the transcriptional activity of NF-κB in a Nur77-dependent manner (Fig. 2), thus effectively activating Nur77’s anti-inflammatory function [29].

## PHENOTYPE-BASED/FORWARD CHEMICAL BIOLOGY

Following the establishment of a chemical library and active compound screening, forward chemical biology calls for the development of powerful methods for the identification of one or a few cellular targets from the complex mixture of biomolecules present in cells, including proteins, nucleic acids, carbohydrates and lipids [30–33]. Thus, the search for cellular targets may well be regarded as a quest for a needle in a giant haystack.

### Cell-based screening

With the advantages of high throughput and low costs, cell-based screening assays have been widely used in chemical biological investigation and drug discovery. Cells most used in such an assay are different cancer cell lines or primary cells directly derived from patients. Also, the merging of *in vitro* three-dimensional tissues in organoids presents great potential in compound screens and drug discovery [34]. The phenotypes used in the cell-based assay can refer to the hallmarks of cancer; cytotoxicity, cell cycle, differentiation, invasion and migration, and drug resistance are main test indicators used in cell-based screening assays. However, for high-throughput screening it is better and more appropriate for phenotypic changes to be recorded by reporter gene activity, fluorescence signal or imaging methods. For example, rapid tumor growth leads to excessive oxygen demands and a hypoxic environment in most solid tumors, which adapt to the hypoxic environment by upregulating the transcription of target genes that regulate cell proliferation, angiogenesis, energy metabolism, apoptosis resistance and metastasis. These processes are generally associated with the transcriptional factor HIF-1, which is upregulated in the tumor and is a promising target for cancer chemotherapy [35,36]. Although no HIF-1 inhibitor is clinically available to date, a lot of effort has been made over the last decade to find potent HIF-1 inhibitors, as recently reviewed by Bhattarai *et al.* [37].

Here, we give examples of reporter gene activity-guiding phenotypic screening based on Wnt/β-catenin signaling. Wnt/β-catenin signaling is a highly conserved pathway in organism evolution and regulates many biological processes, and its aberrant activation is closely related to tumor progression [38]. For example, the pathway is frequently activated in colorectal cancer (CRC) as a result of the mutation of adenomatous polyposis coli (APC). In a screen of FDA-approved drugs using the Top-flash (Wnt/β-catenin pathway-responsive firefly luciferase) assay, nitazoxanide (NTZ, Fig. 1), a clinically approved antiparasitic drug, was shown to block Wnt/β-catenin signaling *in vitro* and in a murine model for familial APC, Apc^min/+^ mice, which spontaneously generate tumors. They also showed that NTZ promotes β-catenin degradation independently from glycogen synthase kinase 3β (GSK3β) or APC activity [39]. With a similar screening system, L. Li’s group at the Shanghai Institute of Biological Science of the Chinese Academy of Sciences identified 15-oxospiramilactone (NC043), a diterpenoid derivative, as being able to inhibit Wnt3a or LiCl-stimulated Top-flash reporter activity in HEK293T cells and the growth of CRC, SW480 and Caco-2 cell, together with decreased mRNA and/or protein expression of the Wnt target genes Axin2 and cyclin D1. Further, NC043 did not affect the cytosolic–nuclear distribution and protein level of soluble β-catenin, but decreased β-catenin/TCF4 association in SW480 cells [40]. More recently, they showed that NC043 directly binds to CARF (the collaborator of ARF) through the formation of a covalent bond with the Cys^516^ residue of CARF, and disrupts the CARF-Disheveled (Dvl) interaction, thereby promoting Wnt signaling activation [21]. This group also screened a synthetic chemical library of lycorine derivatives, and identified the small-molecule compound HLY78 as an activator of the Wnt/β-catenin signaling pathway in a Wnt ligand-dependent manner. HLY78 targets the DIX (Dishevelled axin) domain of axin and potentiates the axin-LRP6 (low-density lipoprotein receptor-related protein 6) association, thus promoting LRP6 phosphorylation and Wnt signaling transduction [41].

### Animal-based screening

Compared with cell models, animal models have the advantage of high relevance to disease, because they can mimic pathophysiological features that are similar to those of patients. Although low-throughput, high cost and time-consuming, animal-based screening is indispensable for preclinical research into the efficacy, side effects and toxicity of novel potential drugs. Small animals such as zebrafish, *Drosophila* and *Xenopus* tadpoles are mostly used for the animal-based screening. Using a *Drosophila* Ras-driven tumor model for large-scale screening of 2000 compounds, the glutamine analog acivicin was finally identified as a potent and specific inhibitor of *Drosophila* tumor formation [42]. After screening 26 400 molecules in a T cell-reporting zebrafish model, a novel molecule named lenaldekar was identified as having selective toxicity against leukemia [43]. The compound had similar effects on human leukemia samples and xenograft mice, indicating high relevance between zebrafish and humans. Kalin *et al*. [44] employed *Xenopus* tadpole embryos as an *in vivo* model to identify novel compounds involved in angiogenesis and lymphangiogenesis through a simple phenotypic readout (edema formation or larval lethality), and identified 32 compounds interfering with blood vascular and/or lymphatic development in *Xenopus* from 1280 bioactive compounds.

### Target identification

Phenotypic screening is often the most straightforward way to discover relevant bioactive compounds, usually with unknown molecular targets. Some researchers may argue that target identification for hits from phenotypic screening is unnecessary if the relevant readout from the phenotypic assay is fairly reliable. However, target identification and validation of bioactive small molecules is an essential, and often decisive step for understanding the mechanism of action of a compound and for further compound optimization [30,45]. Through the development and availability of several new experimental techniques, in principle, target identification is feasible and the number of successful examples is growing steadily, as reviewed by Ziegler *et al*. [46].

Bioactive compounds may interact with off-target proteins, resulting in undesired biological activity or toxicity [47]. We can optimize compounds with more powerful activity and less toxicity by knowing both their therapeutic targets and off-targets. Recent technological advances in genomics, proteomics and bioinformatics have accelerated the process of target identification. Proteomics- and genomics-based approaches provide with powerful tools for all-round identification [48]. Quantitative mass spectrometry (MS) techniques have greatly enhanced the sensitivity of target protein detection [49], while protein microarrays have greatly simplified the process of target identification [50]. Up to now, a number of technologies have been explored to identify targets from phenotypic screens.

Among the techniques currently available in small-molecule target identification, protein affinity isolation, using suitable small-molecule probes (pulldown) and subsequent MS analysis of the isolated proteins, appears to be the most powerful and most extensively used approach, and makes the identification of the full protein-binding spectrum of a compound possible. After a structure–activity relationship (SAR) study, an active compound is conjugated with a specific affinity tag such as polyethylene glycol and biotin at an appropriate position without affecting the compound's activity. Then the compound-tag is incubated with cells or total cell lysates, followed up by the capture of target proteins using a specific solid matrix. Lastly, target proteins are revealed by MS. Although affinity-based approaches can provide an effective method for target identification, it is a challenge to find the appreciate position of active compound for the conjugation of an affinity tag without affecting the compound activity. Besides, the affinity tag somehow alters the structure of the original compound, resulting in false positive and negative targets.

Protein microarrays also provide a high throughput and high sensitive method for the target identification of small molecules. As for this, desired proteins (up to thousands) are immobilized on a treated glass microscope slide to generate a protein microarray [51]. Small molecules need to be labeled with a reporting tag (fluorescence, biotin or isotope), and then they are incubated with the protein microarray, followed up by washing and signal developing. Huang *et al*. [52] identified small-molecule inhibitors and enhancers of rapamycin through a yeast proteome microarray. By using a protein-domain microarray of human methyllysine effector molecules and biotin-labeled UNC1251 analogs, Bae *et al*. [53] identified EML405 as an inhibitor of tudor-domain-containing protein Spindlin 1 (SPIN1). Regardless of the type of labeling, protein microarrays owe the same disadvantage of affinity-based approaches; that is, the labeled small molecule may interfere with the small molecule–protein interaction.

Label-free approaches are relatively simple and direct approaches which do not require any chemical modification of an active compound. This kind of strategy relies on the principle that the protein becomes susceptible to proteolysis once it binds to a small molecule [32,54]. The drug affinity responsive target stability (DARTS) is one of such label-free approaches with the principle that small-molecule binding proteins are protected and enriched during proteolysis, and it has been used to successfully identify cellular targets for several active compounds [31]. After the above-mentioned finding of NTZ for its inhibition of Wnt signaling, for instance, Qu *et al*. [39] profiled proteins that bind directly using DARTS and found that NTZ directly interacts with peptidyl arginine deiminase 2 (PAD2), an enzyme that catalyzes the conversion of the protein arginine residue to citrulline (a post-translational modification (PTM) called deamination or citrullination) and citrullinates β-catenin to promote β-catenin degradation.

### A detailed example for target identification with the needle in the haystack

Leukemia is an aggressive and heterogeneous disorder of malignant hematopoiesis that occurs worldwide. In the past thirty years, many important advances have been achieved in the biological, molecular and cytogenetic aspects of leukemia. It has been widely understood that various kinds of leukemias present specific cytogenetic alterations, especially chromosome translocations, which generate abnormal oncogenic fusion proteins. These alterations disrupt the normal signaling of hematopoietic development and cause uncontrolled proliferation, blocked differentiation and/or damaged apoptosis of malignant hematopoietic cells. In the past years, some natural compounds from TCM and synthetic small compounds were investigated for their antileukemia activity [55–57]. Adenanthin, a kind of ent-kaurene diterpenoids, was originally isolated from the leaves of *Rabdosia adenantha*. The previous investigations demonstrated that diterpenoids have a wide spectrum of biological activities such as antitumor, anti-inflammation and significant cardiovascular effects. Up to now, more than 600 diterpenoids have been found in china. Previously, we also identified pharicin B, a novel natural ent-kaurene diterpenoid derived from *Isodon pharicus* leaves, to rapidly stabilize RARα (*r*etinoic *a*cid *r*eceptor α) protein in various subtypes of AML cells, especially APL, and thus to present a synergistic or additive differentiation-enhancing effect in combination with ATRA [58]. We also found that pharicin A, another new ent-kaurene diterpenoid, induces mitotic arrest in leukemia and solid tumor-derived cells, which is associated with unaligned chromosomes, aberrant BubR1 localization and deregulated spindle checkpoint activation [59]. With the encouragement from these works, we used a cell-based phenotypic assay to screen up to 400 natural ent-kaurene diterpenoids which were provided and isolated by H. D. Sun’s group in Kunming Institute of Botany of Chinese Academy of Sciences [60]. Among these diterpenoids, adenanthin was shown to decrease viability of APL cells at a concentration of more than 4 μmol, while it also induce the differentiation of APL cell lines and primary leukemic blasts from APL and non-APL AML patients [61,62]. Further investigations in ATRA-sensitive and ATRA-resistant APL transgenic mice models revealed that intravenous administration of adenanthin (5 mg kg^−1^ body weight, each day for five consecutive days a week) significantly induces differentiation and tumor regression, and prolongs the survival of both kind of leukemic mice [61]. Moreover, adenanthin also markedly eliminates APL-initiating progenitor cells (CD34^+^, c-kit^+^, FcγRIII/II^+^, and Gr1^int^) in ATRA-sensitive leukemic mice. All these results strongly suggest that adenanthin has potential therapeutic efficacy on AML.

**Figure 3.**
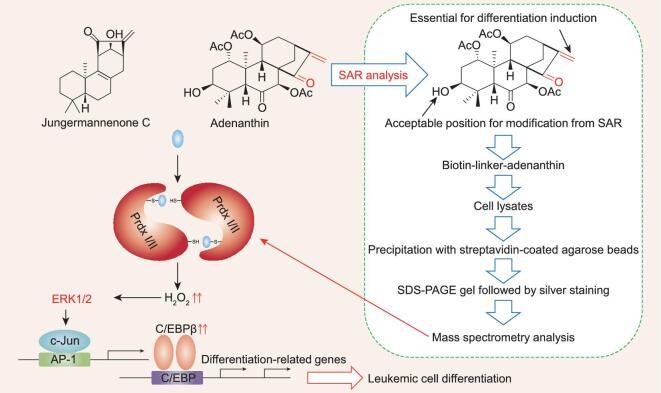
Adenanthin and Jungermannenone C target Prdx I/II to induce leukemic cell differentiation. A flow chart for SAR analysis and target identification of adenanthin is shown in the dash-framed box.

To further study the molecular mechanism of action of adenanthin, we tried to identify its potential protein target(s) via a chemical proteomic approach. After a SAR study of adenanthin, we synthesized a biotin-tagged adenanthin probe without affecting its differentiation-inducing activity. Thus, this biotin-tagged adenanthin probe was applied into the lysates of APL cell line NB4 cells, followed by precipitation with streptavidin-coated agarose beads. The bound proteins were run on an SDS-PAGE gel. On the gel with silver staining, only one detectable band at approximately 23 kD was clearly precipitated by biotin-adenanthin but not by free biotin, and the precipitated band could be competitively inhibited by higher concentrations of unlabeled adenanthin. Finally, MS analysis revealed that the adenanthin-bound protein is a mixture of peroxiredoxin (Prdx) I and Prdx II (Fig. 3) [61,62].

Prdxs are a family of small non-seleno peroxidases that catalyze the peroxide reduction of H_2_O_2_, a reactive oxygen species (ROS), that also play an important role as second messengers in cellular signaling pathways [63,64]. Prdxs have a conserved cysteine named the peroxidatic cysteine (C_P_) that serves as the site of oxidation by peroxides. Peroxides oxidize the C_p_-SH to C_p_-SOH, which reacts with another cysteine typically named the resolving cysteine (C_R_), forming a disulfide bond that is subsequently reduced by an appropriate electron donor. Depending on the location or absence of the C_R_, the mammalian Prdxs are classified into two-cysteine (Prdxs I–IV), atypical two-cysteine (Prdx V) and one-cysteine Prdx (Prdx VI) subfamilies. Thus, we further blotted the precipitates with antibodies against Prdx I–VI, all of which were expressed. The results showed that biotin-adenanthin effectively pulled Prdx I/II down but not Prdxs IV–VI, which was also confirmed by the *in vitro* recombinant Prdxs proteins. Next, we incubated the recombinant Prdx I protein with or without adenanthin followed by MS analysis to determine the adenanthin-modified specific residue, and the results revealed that adenanthin covalently modifies Cys^173^, the C_R_ of Prdx I. Remarkably, adenanthin also selectively bound the C_R_ (Cys^172^) of Prdx II. Accordingly, adenanthin effectively inhibited the peroxidase activity of recombinant Prdx II, and especially Prdx I.

To validate whether targeting Prdx I/II is directly associated with adenanthin-induced differentiation, we knocked down the expression of Prdx I or Prdx II in NB4 cells using specific small interfering RNAs, and found that knockdown of Prdx I or Prdx II induced NB4 cell differentiation. Our further studies showed that adenanthin treatment can moderately increase intracellular H_2_O_2_ level, and that the elevated H_2_O_2_ activates signal-regulated protein kinases 1 and 2 (ERK1 and ERK2), consequently increasing expression of CCAAT/enhancer binding protein-β (C/EBPβ), which has been widely shown to drive AML cell differentiation (Fig. 3) [61].

More intriguingly, recent work from X. G. Lei’s group at Peking University also showed that the natural product jungermannenone C (Fig. 3), a tetracyclic diterpenoid isolated from liverworts, induces AML cells to undergo differentiation through targeting Prdx I/II by selectively binding to the conserved cysteine residues, thus leading to cellular ROS accumulation (Fig. 3) [65]. More intriguingly, a recent report unveiled the biological function of the cyclin-dependent kinase 2 (CDK2)-Prdx II axis in blocking AML differentiation [66]. They showed that CDK2 undergoes ubiquitin-dependent proteasome degradation by the specific E3 ubiquitin ligase KLHL6, which is accompanied by AML cell differentiation. Importantly, inhibiting CDK2 effectively induces granulocytic differentiation in AML cells and the differentiation blockade function of CDK2 may be achieved directly by maintaining the activity of Prdx II [66].

## TARGET-BASED SCREENING/REVERSE CHEMICAL BIOLOGY

In target-based screening approaches, the target of interest is usually recombinantly expressed in a purified system or in a cellular context, and then specific small chemicals with potential ability to modulate the activity of the target are screened through an appropriate *in vitro* assay. This approach is relatively easy to execute, with less cost in terms of time and money compared to phenotype-based screening approaches. However, there are still big challenges, because an active compound identified in an *in vitro* assay may not work *in vivo* due to the complex environment, such as the cell membrane impenetrability of the compound, PTMs of the target, undesired targets and the compound metabolism of the intact organism. In spite of these challenges, many activators or inhibitors of receptors and enzymes have been successfully identified through this screening approach. In particular, small-molecule inhibitors of a number of recently identified protein targets offer new therapeutic options tailored to specific mutations or to counter resistance. Imatinib, the first tyrosine kinase inhibitor to be identified, was discovered to have high specificity for the Bcr-Abl protein that results from t(9, 22)-derived Philadelphia chromosome in chronic myeloid leukemia (CML). However, several Bcr-Abl-dependent and -independent mechanisms of resistance to imatinib arose after it became a first-line therapy in CML patients. Consequently, new specific drugs, such as dasatinib, nilotinib, bosutinib and ponatinib have been rationally designed and approved for clinical use to override the problem of resistance [67]. On the basis of the finding that the NEDD8-activating enzyme subunit NAE1 is overexpressed in CML cells, the NAE1 inhibitor MLN4924 was also demonstrated to induce G_2_-M arrest and apoptosis in CML cells, regardless of their T315I mutation status in Bcr-Abl, which offers a preclinical proof of concept for targeting protein neddylation as a novel therapeutic strategy to override mutation-derived imatinib resistance in CML [68]. In a c-Myc pathway-targeted screen of seven natural anticancer compounds, cryptotanshinone was identified as a dual inhibitor of pSTAT5 and pSTAT3, which effectively blocks IL-6-mediated STAT3 activation and reverse Bcr-Abl kinase-independent drug resistance in CML [69]. In the following section, we will provide several examples of target-based discoveries of active compounds against cancers, including leukemia.

### Pyruvate dehydrogenase kinase 1 inhibition

One of the general hallmarks of malignancy is a unique metabolic profile of high aerobic glycolysis, a phenomenon of the enhanced conversion of glucose into lactate even in the presence of oxygen. The aerobic glycolysis, known as the Warburg effect, confers a significant growth advantage on cancer cells by supplying essential ATP production, generating precursors for biosynthesis and providing reducing equivalents for antioxidant defense. Recently, an increasingly recognized link between oncogenic proteins (for example, HIF-1 or its cooperation with dysregulated c-Myc) and pyruvate dehydrogenase kinase 1 (PDK1), a molecular switch that diminishes mitochondrial respiration and enhances aerobic glycolysis via phosphorylating and inactivating pyruvate dehydrogenase, has provided a glimpse into the molecular basis of the metabolic reprogramming that occurs in cancer cells. To discover new PDK1 inhibitors, M. Y. Geng’s group at the Shanghai Institute of Materia Medica of the Chinese Academy of Sciences carried out a PDK1 enzymatic screen using an in-house small-molecule library composed of ∼600 commercially available known drugs, and thiram, an existing pesticide with anticancer activity, was found to be capable of remarkably inhibiting PDK1 activity. Based on thiram, their further chemical efforts led to the discovery of a more potent new compound designated as bis(4-morpholinyl thiocarbonyl)-disulfide (JX06), which was identified as a selective covalent inhibitor of PDK1 in cancer cells [70]. JX06 forms a disulfide bond with the thiol group of a conserved cysteine residue (C^240^), based on its recognition of a hydrophobic pocket adjacent to the ATP pocket of the PDK1 enzyme. With the covalent modification at C^240^, conformational changes at Arg^286^ through van der Waals forces are induced, thereby hindering access of ATP to its binding pocket and, in turn, impairing PDK1 enzymatic activity. Notably, cells with a higher dependency on glycolysis were more sensitive to PDK1 inhibition [70], reflecting a metabolic shift that promotes cellular oxidative stress and apoptosis.

### IDH1 inhibition

Point mutations affecting isocitrate dehydrogenase 1 (IDH1) Arg^132^ (R132), and IDH2 Arg^172^ or Arg^140^ (R172 or R140), are driver mutations in AML and other cancers. A high-throughput biochemical screen targeting an IDH1 heterodimer composed of R132H mutant IDH1 and wild-type IDH1 identified a tetrahydropyrazolopyridine series of inhibitors, and additional structure optimization led to the identification of GSK321 as a highly potent inhibitor of mutant IDH1 enzymes, with IC_50_ values of 4.6 nM against R132H, 3.8 nM against R132C and 2.9 nM against R132G. GSK321 binds to an allosteric site and locks the IDH1 enzyme in a catalytically inactive conformation, thereby enabling the inhibition of different clinically relevant IDH1 mutants. GSK321 treatment of primary AML cells with mutant IDH1 uniformly led to the induction of granulocytic differentiation at the level of leukemic blasts and more immature stem-like cells, *in vitro* and *in vivo*, together with a decrease in intracellular oncometabolite 2-hydroxyglutarate [71].

### SIRT6 activation

One PTM is lysine side chain N^ϵ^-acylation, which can be reversed through the deacylation of the resulting N^ϵ^-acyl-lysine. The sirtuins, which have seven sirtuin members (SIRT1–7), are able to catalyze the Nϵ-acyl-lysine deacylation reaction on histone and are a type of acyltransferase, thus can catalyze the removal of the acyl group from lysine residues on histones and other non-histone protein substrates. The sirtuin-catalyzed deacylation reaction plays an important regulatory role in multiple crucial cellular processes such as transcription, DNA damage repair, genomic stability, cell cycle, apoptosis, inflammation, metabolism and caloric restriction. The reaction is also regarded as a current therapeutic target for human diseases such as cancers. More recently, a variety of chemical probes and modulators (inhibitors and activators) have been developed, and some of them have been employed toward an enhanced mechanistic and functional understanding of the sirtuin-catalyzed deacylation reaction [72].

SIRT6, one of the most important sirtuin family members, which is widely expressed in almost all mammalian tissues, has been implicated in regulating several biological processes, including DNA repair, glucose/lipid metabolism, inflammation and aging. A series of studies have revealed that SIRT6 functions as a tumor suppress gene [73,74], but there are still no small-molecule activators specific to SIRT6 that have been found to date. We hypothesized that SIRT6 can be activated via an allosteric mechanism. Thus, we adopted the allosite method [75] to predict allosteric sites for the activation of SIRT6, resulting in a pocket around Phe^82^ and Phe^86^ being identified as a potential site. After virtual docking with more than 5 000 000 compounds, the top-ranked 20 compounds were selected out for experimental validation. Two hits were confirmed to be able to increase the activity of SIRT6 deacetylation in the Fluor-de-Lys assay. Subsequent optimization based on these two hits yielded two potent activators: MDL-800 and MDL-801, with EC_50_ values of 10.3 ± 0.3 μM and 5.7 ± 0.023 μM, respectively. MDL-800 was shown to be a selective activator of SIRT6 among the 18 diverse histone deacetylase members. They also showed that MDL-800 can arrest the cell cycle of human hepatocellular carcinoma (HCC) cells and inhibit HCC in a xenograft mice model by enhancing the activity of SIRT6 deacetylation [76].

### Speckle-type POZ protein inhibition

BTB (BR-C, ttk and bab) domain-containing speckle-type POZ protein (SPOP), an adaptor of ubiquitin E3 ligase, plays important roles in development and tumorigenesis by mediating the ubiquitination of multiple substrates, such as phosphatase and tensin homolog (PTEN) and dual specificity phosphatase 7 (DUSP7). Previous work showed that SPOP is overexpressed in virtually all clear cell renal cell carcinomas (ccRCCs), which accounts for about 75% of all RCC cases [77], and that overexpressed SPOP, which is a nucleoprotein in normal cells, accumulates in the cytoplasm of ccRCC cells. Furthermore, SPOP serves as a regulatory hub to promote ccRCC tumorigenesis through the ubiquitination and degradation of multiple regulators of cellular proliferation and apoptosis. These ideal characteristics make SPOP a potential antitumor target for the treatment of ccRCC. Based on structural studies, Jiang’s group at the Shanghai Institute of Materia Medica of the Chinese Academy of Sciences performed computational screening through a hierarchical strategy combining pharmacophore modeling and molecular docking, and 109 compounds were selected from the SPECS database with ∼200 000 drug-like structures [78]. They first performed a fluorescence polarization (FP) assay to measure the ability of small molecules to competitively inhibit SPOP-binding consensus (SBC1) peptide *in vitro*. After compound 6a was identified as an initial hit capable of competing with SBC1 peptide binding to SPOP, additional synthetic optimization of the chemical core successfully yielded the more active lead compound 6b with a K_d_ value of 35 μM on FP measurement. The *in vitro* pull-down assay showed that compound 6b obviously disrupts the interaction of SPOP with its substrate PTEN protein *in vitro* with an IC_50_ of 2.8 μM, and a co-immunoprecipitation experiment showed that the inhibitor significantly disrupts SPOP binding to PTEN and DUSP7 in a dose-response manner. Accordingly, the compound efficiently inhibited the growth of six ccRCC cell lines and all primary ccRCC cells isolated from seven patients, with minimal effect on the growth of one non-tumor human proximal tubule epithelial cell line HK-2. Furthermore, a clear dose-dependent reduction in A498 tumor growth rate could be observed in mice treated with compound 6b compared with the vehicle-injected control [78]. These results imply that SPOP cannot be viewed as classically ‘undruggable’, and opens up the avenue that small molecule-targeting cytoplasmic SPOP signaling might be more specific to ccRCC cells, which would be a promising strategy to combat kidney cancer in future therapies.

### PPI inhibition

With an estimated 650 000 PPIs as part of the human interactome, PPIs are a critical means for the majority of proteins to exert and regulate their functions, and play crucial roles in various cellular processes and signaling pathways. Rapid advances in MS technology have allowed protein interactions to be elucidated in a systematic manner, and have tremendously enhanced our understanding of biological pathways and networks [79]. Dysregulation in PPIs is often found to be the primary cause of the pathogenesis of some diseases, especially cancers. Thus, PPIs have become popular and attractive therapeutic targets. Although the development of PPI modulators (inhibitors or stabilizers) has been hindered because of the seemingly low druggability of PPI interfaces, extensive studies of PPI targets and modulators have been performed to better understand these complex targets and identify distinct properties in their networks, conformational structures and ligand chemical spaces. Also, some PPI modulators have entered clinical trials or clinical use, as summarized in a recent review by Macalino *et al*. [80], which have ascertained that these targets are tractable and can be modulated by small-molecule compounds. Here, we provide three recent examples of discoveries of target-based compounds against cancers.

#### Mixed lineage leukemia/menin interaction inhibitor

Mixed lineage leukemia (MLL) is a common target of chromosomal translocations found in patients with AML and ALL. Its fusion with one of over 50 different partner genes forms chimeric oncogenes encoding MLL fusion proteins, in which the N-terminal 1400-amino acid fragment of MLL is preserved and fused to distinct protein partners. The leukemogenic activity of MLL fusion proteins is critically dependent on their direct interaction with menin, a product of the multiple endocrine neoplasia (MEN1) gene. Grembecka *et al*. [81] screened a collection of 49 000 small molecules using an FP assay with a fluorescein-labeled MLL-derived peptide comprising the high-affinity menin-binding motif, MBM1, to identify initial lead compounds that target menin and inhibit the menin-MLL interaction. They found that the most potent compound, MI-1, which belongs to the thienopyrimidine class, reversibly inhibits the menin-MLL interaction with an IC_50_ value of 1.9 μM. The compound effectively reverses MLL fusion protein-mediated leukemic transformation by downregulating the expression of target genes, selectively blocks proliferation, and induces both the apoptosis and differentiation of leukemia cells harboring MLL translocations.

#### CBFβ-SMMHC/RUNX1 interaction inhibitor

AML with the chromosome inversion inv(16)(p13q22), a driver mutation that generates preleukemic progenitor cells that, upon acquisition of additional cooperating mutations, progress to leukemia, expresses the transcription factor fusion CBFβ-SMMHC (core binding factor β-smooth muscle myosin heavy chain), which cooperates with activating mutations in components of cytokine signaling pathways in leukemia transformation. CBFβ is a component of the heterodimeric transcription factor core binding factor, where it binds to RUNX proteins and enhances their affinity for DNA, and the resulting complex plays a key role in regulating hematopoiesis. The CBFβ-SMMHC outcompetes wild-type CBFβ for binding to the transcription factor RUNX1, deregulates RUNX1 activity in hematopoiesis, and induces AML. Illendula *et al*. [82] used a fluorescence resonance energy transfer (FRET) assay to screen compounds that inhibit the binding of CBFβ-SMMHC to the RUNX1 Runt domain, and identified the active compound AI-4-57, which has an IC_50_ of 22 μM. Based on the lead compound, they found that AI-10-49 selectively binds to CBFβ-SMMHC and disrupts its binding to RUNX1, which thus restores RUNX1 transcriptional activity, displays favorable pharmacokinetics and delays leukemia progression in mice. These data suggest that direct inhibition of the oncogenic CBFβ-SMMHC fusion protein may be an effective therapeutic approach for inv(16) AML, and they provide support for transcription factor-targeted therapy in other cancers.

#### APC-Asef interaction inhibitor

**Figure 4.**
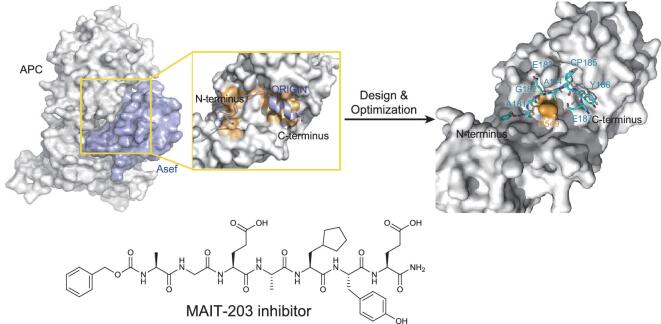
Design and optimization of a small peptide to inhibit APC-Asef interaction.

Mutation and inactivation of APC, a widely accepted tumor suppressor gene highly mutated in CRC, is a key and early event that is almost uniquely observed in colorectal tumorigenesis. As a multidomain protein, APC serves multiple functions through different binding partners. From the N-terminus to the C-terminus, there is an oligomerization domain, an armadillo repeat domain, a 15- or 20-residue repeat domain, a SAMP (Ser-Ala-Met-Pro) repeat domain, a basic domain and a C-terminal domain. Alterations in the APC gene generate truncated gene products, leading to activation of the Wnt signaling pathway and deregulation of multiple other cellular processes. APC mutant proteins that retain at least the first 171 amino acids are able to bind to the oligomerization domain and may have a dominant negative effect on the APC protein. The armadillo repeat domain is the most conserved domain and has been shown to bind to IQ motif-containing GTPase activation protein 1 (IQGAP1), PP2A, Asef (also known as Rho guanine nucleotide exchange factor 4) and KAP3. APC-Asef interaction can relieve the negative intramolecular regulation of Asef, which leads to aberrant migration in human CRC, suggesting that this interaction might be a potential target for the treatment of invasive migration in colorectal cancer. To identify potent inhibitors of APC-Asef interaction, we analyzed the direct binding interface on the basis of the previously determined structure of the APC-Asef complex, and a hot spot (^181^GGEQLAI^187^) in a flexible segment of Asef was used to design inhibitors (Fig. 4) [83]. Then, a series of truncated peptides were synthesized and verified through an FP competitive assay. MAI-005 was identified as the best inhibitor in the first round of screening, with a *K*_i_ value of 44.62 ± 0.99 μM. After the first optimization with mutations at each position of MAI-005, three peptides were obtained with more potency (*K*_i_ values are 3.12 ± 0.70 μM, 3.80 ± 0.72 μM and 2.41 ± 0.88 μM, respectively). From analysis of the structures of APC in complex with each of the three peptides, Arg549 at the center of the APC pocket was shown to have increased potential for interactions with the C-termini of peptide inhibitors through salt-bridge or hydrogen bonds. Based on this, a second peptide library was generated that contains restricted polar or acidic diversity at position 187, fully randomized diversity at position 186 and various capping groups on the terminus. MAI-150 was identified as the most potent peptide with a *K*_i_ value of 0.12 ± 0.02 μM through the FP assay. For further optimization of MAI-150, we synthesized a panel of more than 50 peptidomimetic inhibitors, optimized for a capping group, as well as the Leu185 and Tyr186 side chains. Finally, MAI-203 (Fig. 4) was identified as the most potent peptide with a *K*_i_ value of 0.015 ± 0.001 μM through the FP assay. Next, it was examined whether MAIs inhibit APC-Asef interaction *in vivo*. An optimized ‘GGGGG’ linker was conjugated to the C-terminus of the inhibitors (referred as MAITs relatively) to facilitate their movement across the cell membrane. A co-immunoprecipitation assay was carried out in HEK293T cells to corroborate the inhibitory effect of MAITs on APC-Asef interaction *in vivo*. Incubating cells with MAIT-203 and MAIT-150 reduced APC-Asef interaction in a dose-dependent manner, while incubating with DMSO, a ‘GGGGG’ linker, MAI-150, or MAI-203 showed no effect on APC-Asef interaction, confirming the fact that MAITs inhibit APC-Asef interaction *in vivo*. Through xCELLigence system Real Time Cell Analysis (RTCA), wound-healing assays and transwell assays, we showed that MAIT-203 at 10 μM significantly inhibited the migration of two CRC cell lines (SW480 and HCT116). High concentrations (up to 100 μM) of MAIT-203 did not affect the morphology or growth of SW480 and HCT116 cells, indicating that the antimigratory effect of MAIT-203 works through the disruption of the APC-Asef interaction, rather than cell growth. Besides, the RTCA invasion assay showed that MAIT-203 at 10 μM significantly repressed the invasion of SW480 and HCT116 cells; together with the results of the migration assays, this suggests that MAIT-203 can inhibit the metastasis of CRC cells [83]. These results not only demonstrate the feasibility of exploiting the APC-Asef interaction as a target against metastatic CRC for drug discovery, but also establish a new pharmacological paradigm for the use of peptides/peptidomimetics as inhibitors of protein–protein interactions.

## DEVELOPMENT OF CHEMICAL TOOLS

Much of our mechanistic knowledge about cellular processes has been gained by recreating systems *in vitro*, or by engineering a cell or a model organism such as yeast, nematodes or mice. The chemical toolset needed to probe and validate such models in their real settings historically requires the contribution of chemical biology to biological and biomedical sciences, as addressed by Jiang *et al*. [84]. Therefore, exploring and developing the new chemical toolset is very important for the application of chemical biology to manipulate biological systems. Here, we would like to outline several new strategies for chemistry to probe cellular activities.

### Proximity-dependent labeling methods for PPI identification

As mentioned above, PPIs play important roles in signaling pathways and cellular activities. PPIs can be either static in intact entities or be dynamically regulated. Previously, a variety of biochemical and/or high-throughput screening methods were developed to investigate PPIs *in vitro* or in living cells, such as yeast two-/three-hybridization, phage display, affinity pulldown coupled with MS characterization, protein array, FRET or bioluminescence resonance energy transfer, and others. However, all these methods have some defects, such as presenting high false-positive rates, and failing to perform real-time and *in vivo* PPI analysis. Over the past few years, several groups have independently developed a class of methods termed proximity-dependent labeling (PDL) for PPI mapping. Their basic strategy is that the protein of interest is genetically fused to a proximity-based labeling enzyme such as engineered biotin ligase or peroxidase, both of which are capable of covalently attaching known reactive groups to nearby proteins [85]. In the presence of biotin or biotin-containing substrates, the fused enzyme will activate and then release substrates to label proximal proteins. Interacting proteins that are in close proximity to the protein of interest are more likely to be labeled by the proximity enzyme [86]. Among these PDL methods, BioID (proximity-dependent biotin identification) is the earliest and most widely used. This technique harnesses a promiscuous biotin protein ligase BirA protein from *Escherichiacoli* (for the prototypic BioID) or its R118G mutant named BirA* to biotinylate proteins based on proximity. The ligase is fused to a protein of interest and expressed in cells, where it biotinylates proximal endogenous proteins (for detail, see Roux *et al*. [87]). Recent studies showed that BioID can identify weaker and/or transient interactions, is amenable to temporal regulation, and in particular can be applied to insoluble proteins and a variety of cell types from diverse species. Like BioID, PDL with ascorbate peroxidase (APEX) is active in living cells, and can catalyze biotin-phenol and H_2_O_2_ to generate biotin-phenoxyl radicals that covalently react with specific amino acids. APEX has also been successfully applied to PPI analyses in living cells, as recently summarized by Zhu *et al*. [85]. Recently, Wang and Zhuang’s groups in the School of Life Science and Technology, ShanghaiTech University developed a new PDL system named PUP-IT (pupylation-based interaction tagging) to identify membrane protein interactions, in which a small protein tag, Pup (a small bacterial protein that carries 64 amino acids with Gly-Gly-Gln at the C-terminus), is applied to proteins that interact with PafA, a gene that encodes Pup ligase-fused protein, enabling transient and weak interactions to be enriched and detected by MS. With this approach to CD28, they identified multiple potential CD28-interacting proteins besides its known binding partners. They also showed that this method can identify the cell surface receptor and its ligand interactions [88].

### Genetically encoded sensors for NAD(P)H

Real-time tracing of cell metabolism is a technical bottleneck for biomedical and bioengineering research. Traditional approaches to assess the cellular metabolic state, e.g. biochemical analysis, MS or nuclear magnetic resonance, are not effective for *in vivo*, real-time, spatiotemporal tracing of cellular metabolites. In recent years, Yang and co-workers in the East China University of Science and Technology have contributed significantly to the development of genetically encoded sensors for NADH/NADPH, key metabolites for redox and energy metabolism. Reduced NADH and NADPH are the most important electron carries in cells. They participate in numerous metabolic redox reactions of organisms, and are important parameters for cellular metabolic imbalances and disease states. By rational design of fusion between fluorescent proteins and NADH binding of the bacterial repressor Rex, Yang *et al*. created a series of highly responsive and highly specific genetically encoded sensors for NADH and NADPH, which couple the emitting states of the fluorescent protein chromophore to the conformational changes induced by specific interaction between Rex protein and nicotinamide adenine nucleotide ligands. For instance, the NADH sensor Frex specifically detects free NADH levels in the cells [89], SoNar reports the NADH/NAD ratio [90] and iNap (a rational design mutant of SoNar) specifically detects NADPH [91]. These sensors can be targeted to various subcellular compartments or living tissues by genetic manipulation, which allows dynamic monitoring and imaging of cellular metabolic states in living cells or *in vivo*. Considering the central roles that NADH and NADPH play in cell metabolism and signaling, dynamic and quantitative monitoring of NADH and NADPH levels *in situ* is not only useful to the better understanding of substance and energy metabolism regulation and networks, but also provides useful tools for drug biosynthesis, metabolic engineering and drug discovery for metabolic diseases. These technological advances have garnered a lot of attention from international peers, and are currently being applied to various model organisms including bacteria, yeasts, fruit flies and mammals. Due to their favorable characteristics, these sensors are anticipated to revolutionize the study of redox biology, as many unresolved questions regarding the regulation and physiological roles of different redox couples can be directly addressed.

### LightOn and LightOff gene expression

Regulation of gene expression is of vital importance for the study of various life activities such as cell metabolism. Gene expression systems induced by small molecular chemicals such as tetracycline are not able to precisely control gene expression spatially and in a single cells. Based on the photosensitivity of the riboflavin moiety in the light-oxygen-voltage-sensing domain, LightOn was developed as a light-activatable transcription activator, and a robust and convenient single-component light-switchable gene expression system. LightOn enables spatiotemporal, quantitative and reversible expression regulation of functional genes such as insulin and Cre recombinase *in vivo*, and can control blood glucose levels and glucose metabolism in diabetic mice [92]. Further studies showed that integration of the system with a tetracycline-inducible gene expression system led to gene expression only in the presence of inducer and light, which is significantly more stringent [93]. Based on similar strategies, the group also developed a light-activated repressor, the LightOff gene expression system. This system has achieved extremely stringent regulation of gene expression with an induction ratio greater than 10 000-fold, far exceeding that of chemically inducible gene expression systems [94]. These novel gene expression systems provide powerful tools not only for the analysis of complex biological systems, but also for accurate therapeutic strategies of accurate timing and dosage for important human diseases such as diabetes. For example, the LightOn system offers an efficient way to control the proliferation and differentiation of neural progenitor cells by changing the light-exposure pattern, showing its applicability to regeneration technology [95].

### Bioorthogonal cleavage reactions for gain-of-function studies of proteins

Cells have evolved a rich repertoire of enzymes to catalyze potent chemical modifications on diverse proteins that dictate virtually all signaling events. For example, nearly 600 kinases exist in human cells that control phosphorylation, and this enzymatic network is complicated further by the presence of many phosphatases that catalyze the removal of phosphorylation to reverse the signaling. Misregulation of these enzymes is often linked with various diseases such as cancer and inflammation. Meanwhile, oncogenic mutations on these signaling proteins are common features of cancers. Moreover, this process is further complicated by the feedback regulation of enzyme and signaling protein networks, and little is known about this critical ‘fine-tuning’ mechanism. *In vivo* manipulation of a given enzyme with high specificity and spatiotemporal resolution is highly desirable, yet exceedingly difficult to achieve. To address these challenges, the bioorthogonal cleavage reaction-based ‘chemical decaging’ strategy has been developing and thriving in recent years, as a chemistry-enabled strategy to label and manipulate biomolecules without the interference of native biochemical processes. For instance, the critical roles of diverse PTMs on proteins have been increasingly appreciated, with many of the underlying mechanisms hardly addressable by traditional genetic-based approaches. An array of bioorthogonal chemistry tools have been developed to track, visualize and modify proteins, as well as other biomolecules under living conditions, which has significantly facilitated the study of these PTM events. However, almost all previous efforts have been centered on ‘bond-formation’ reactions. In the opposite direction, Chen’s group at Peking University started to recognize a need to develop bioorthogonal ‘bond-cleavage’ tools that could be applied for spatiotemporal controlled rescue of intracellular proteins, surface glycans and even intact cells within a native cellular environment [96]. They showed that simple palladium species can effectively catalyze the depropargylation reaction with low toxicity in living cells, which can be employed as a biocompatible chemical decaging strategy to rescue intracellular protein activity [97]. They also employed the inverse electron demand Diels–Alder (iDA) reaction as a small molecule-triggered bioorthogonal cleavage reaction. The classical iDA reaction has been used as a ‘bioorthogonal-triggered release’ strategy via installation of a carbamate group next to the double bond on trans-cyclooctene (TCO), which readily reacts with tetrazines followed by rearrangement through electron shift, resulting in the deprotection of the TCO group from the protected amine moiety. They have elegantly demonstrated the application of this reaction as a chemical decaging strategy on essential lysine residues on various proteins [98].

By coupling these bioorthogonal cleavage reactions with the genetic code expansion methodology, they have also created a mechanism-based kinase-activation strategy [99]. The activity of each of the near 600 kinases is precisely regulated by their native physiochemical inputs, which are often entangled within the complicated signaling networks, making it exceedingly difficult to manipulate a single kinase with high specificity and spatiotemporal resolution. Gain-of-function studies of kinases are advantageous in probing the sufficiency of a specific kinase, as opposed to the more widely adopted loss-of-function methods, but a general ‘activation’ tool is lacking. Their kinase decaging strategy relies on replacing the kinase's catalytic lysine with a chemical-caged lysine analog, TCOK, via genetic code expansion to blockage of its enzymatic activity. The subsequent addition of the bioorthogonal cleavage trigger Tetrazine unmasks this lysine and thus rescues the corresponding native kinase. They have applied this strategy to specifically rescue a panel of kinases such as mitogen-activated protein kinase kinase-1/2, Focal adhesion kinase (FAK) and Src in living cells, and have further extended its utility to living animals [99]. In sum, these approaches offer a general tool to rescue the native sequences of desired protein machineries *in situ* such as PTM enzymes, metabolic enzymes and epigenetic regulators, which are uniquely positioned for gain-of-function, in contrast to more conventional loss-of-function studies of proteins within living systems.

## CONCLUSION

The field of cancer biology has long benefited from the mechanistic insights provided by appropriately characterized chemical probes or tools of sufficient quality. These chemical probes have enabled the spatiotemporal study of cellular pathways to interrogate complex biological systems. Numerous breakthroughs in biology have been enabled by the use of chemical probes or tools of sufficient quality, especially in combination with complementary biological reagents and molecular technologies [5]. In fact, the great reservoir of natural compounds derived from higher plants, such as TCM herbs, has played an important role in the discovery and development of new chemical probes, and also therapeutic drugs. The number of different species of plants all over the world is enormous (ranging from 215 000 to 500 000). However, to date, only about 6% and 15% of these have been screened for biological activity, and evaluated phytochemically, respectively. Therefore, we should continue to explore these small molecules, particularly those with well-defined biological potency, selectivity and cell permeability. In particular, China should provide financial and policy support for the collection of chemical compounds in nation-level libraries to be shared by all scientists. On the other hand, chemists and biologists should join together to further explore and develop strategies of target identification and validation. In the era of big data especially, artificial intelligence technology should also be developed in various fields of chemical probe, and drug design and target identification [100,101]. Finally, there are challenges to be overcome in the selection and use of chemical probes. The criteria for high-quality chemical probes often need to be more stringent than those for drugs used in patients, especially with regard to selectivity, because chemical probes need to be much more selective to investigate specific biological questions. Therefore, careful chemical optimization and biological testing must be carried out to minimize the risk of off-target effects in chemical probes.

## References

[1] Altmann KH , Buchner J, Kessler H et al. The state of the art of chemical biology. ChemBioChem 2009; 10: 16–29. 19115274 10.1002/cbic.200800758

[2] Bucci M , Goodman C, Sheppard TL. A decade of chemical biology. Nat Chem Biol 2010; 6: 847–54. 21079586 10.1038/nchembio.489

[3] Filippakopoulos P , Qi J, Picaud S et al. Selective inhibition of BET bromodomains. Nature 2010; 468: 1067–73. 20871596 10.1038/nature09504 PMC3010259

[4] Nicodeme E , Jeffrey KL, Schaefer U et al. Suppression of inflammation by a synthetic histone mimic. Nature 2010; 468: 1119–23. 21068722 10.1038/nature09589 PMC5415086

[5] Blagg J , Workman P. Choose and use your chemical probe wisely to explore cancer biology. Cancer Cell 2017; 32: 9–25. 28697345 10.1016/j.ccell.2017.06.005 PMC5511331

[6] Tang YJ , Gholamin S, Schubert S et al. Epigenetic targeting of Hedgehog pathway transcriptional output through BET bromodomain inhibition. Nat Med 2014; 20: 732–40. 24973920 10.1038/nm.3613 PMC4108909

[7] Kostic M , Crews CM, Hertweck C et al. Voices of chemical biology: charting the next decade. Cell Chem Biol 2016; 23: 199. 26971865 10.1016/j.chembiol.2016.02.006

[8] Jiang H , Wu J, Zhang L et al. Chemical biology in China takes on signal transduction. Nat Chem Biol 2008; 4: 515–8. 18711374 10.1038/nchembio0908-515

[9] Zhang XW , Yan XJ, Zhou ZR et al. Arsenic trioxide controls the fate of the PML-RARalpha oncoprotein by directly binding PML. Science 2010; 328: 240–3. 20378816 10.1126/science.1183424

[10] Li K , Wang F, Cao WB et al. TRIB3 promotes APL progression through stabilization of the oncoprotein PML-RARalpha and inhibition of p53-mediated senescence. Cancer Cell 2017; 31: 697–710.e7. 28486108 10.1016/j.ccell.2017.04.006

[11] Hou P , Li Y, Zhang X et al. Pluripotent stem cells induced from mouse somatic cells by small-molecule compounds. Science 2013; 341: 651–4. 23868920 10.1126/science.1239278

[12] Yang Y , Liu B, Xu J et al. Derivation of pluripotent stem cells with in vivo embryonic and extraembryonic potency. Cell 2017; 169: 243–57.e25. 28388409 10.1016/j.cell.2017.02.005 PMC5679268

[13] Li X , Yang Y, Yang Z et al. Chemical reprogramming: the CiPSCs and the CiNs. Natl Sci Rev 2017; 4: 7–10.

[14] Workman P , Al-Lazikani B. Drugging cancer genomes. Nat Rev Drug Discov 2013; 12: 889–90. 24287764 10.1038/nrd4184

[15] Blagg J , Workman P. Chemical biology approaches to target validation in cancer. Curr Opin Pharmacol 2014; 17: 87–100. 25175311 10.1016/j.coph.2014.07.007

[16] Shen Q , Cheng F, Song H et al. Proteome-scale investigation of protein allosteric regulation perturbed by somatic mutations in 7,000 cancer genomes. Am J Hum Genet 2017; 100: 5–20. 27939638 10.1016/j.ajhg.2016.09.020 PMC5223033

[17] Patel MN , Halling-Brown MD, Tym JE et al. Objective assessment of cancer genes for drug discovery. Nat Rev Drug Discov 2013; 12: 35–50. 23274470 10.1038/nrd3913

[18] Guo J , Yu W, Su H et al. Genomic landscape of gastric cancer: molecular classification and potential targets. Sci China Life Sci 2017; 60: 126–37. 27460193 10.1007/s11427-016-0034-1

[19] Newman DJ , Cragg GM. Natural products as sources of new drugs from 1981 to 2014. J Nat Prod 2016; 79: 629–61. 26852623 10.1021/acs.jnatprod.5b01055

[20] Galloway WRJD , Isidro-Llobet A, Spring DR. Diversity-oriented synthesis as a tool for the discovery of novel biologically active small molecules. Nat Commun 2010; 1: 1–13. 20865796 10.1038/ncomms1081

[21] He XL , Zhang WJ, Yan C et al. Chemical biology reveals CARF as a positive regulator of canonical Wnt signaling by promoting TCF/beta-catenin transcriptional activity. Cell Discov 2017; 3: 17003. 28417011 10.1038/celldisc.2017.3 PMC5387711

[22] Jones LH , Bunnage ME. Applications of chemogenomic library screening in drug discovery. Nat Rev Drug Discov 2017; 16: 285–96. 28104905 10.1038/nrd.2016.244

[23] Liu YR , He Y, Yang FF et al. A novel synthetic small molecule YF-452 inhibits tumor growth through antiangiogenesis by suppressing VEGF receptor 2 signaling. Sci China Life Sci 2017; 60: 202–14. 28194552 10.1007/s11427-016-0369-6

[24] Wilson MR , Zha L, Balskus EP. Natural product discovery from the human microbiome. J Biol Chem 2017; 292: 8546–52. 28389564 10.1074/jbc.R116.762906 PMC5448083

[25] To SK , Zeng JZ, Wong AS. Nur77: a potential therapeutic target in cancer. Expert Opin Ther Targets 2012; 16: 573–85. 22537097 10.1517/14728222.2012.680958

[26] Zhan Y , Du X, Chen H et al. Cytosporone B is an agonist for nuclear orphan receptor Nur77. Nat Chem Biol 2008; 4: 548–56. 18690216 10.1038/nchembio.106

[27] Zhan YY , Chen Y, Zhang Q et al. The orphan nuclear receptor Nur77 regulates LKB1 localization and activates AMPK. Nat Chem Biol 2012; 8: 897–904. 22983157 10.1038/nchembio.1069

[28] Wang WJ , Wang Y, Chen HZ et al. Orphan nuclear receptor TR3 acts in autophagic cell death via mitochondrial signaling pathway. Nat Chem Biol 2014; 10: 133–40. 24316735 10.1038/nchembio.1406

[29] Li L , Liu Y, Chen HZ et al. Impeding the interaction between Nur77 and p38 reduces LPS-induced inflammation. Nat Chem Biol 2015; 11: 339–46. 25822914 10.1038/nchembio.1788

[30] Schenone M , Dancik V, Wagner BK et al. Target identification and mechanism of action in chemical biology and drug discovery. Nat Chem Biol 2013; 9: 232–40. 23508189 10.1038/nchembio.1199 PMC5543995

[31] Lomenick B , Hao R, Jonai N et al. Target identification using drug affinity responsive target stability (DARTS). Proc Natl Acad Sci USA 2009; 106: 21984–9. 19995983 10.1073/pnas.0910040106 PMC2789755

[32] Lomenick B , Olsen RW, Huang J. Identification of direct protein targets of small molecules. ACS Chem Biol 2011; 6: 34–46. 21077692 10.1021/cb100294v PMC3031183

[33] Ziegler S , Pries V, Hedberg C et al. Target identification for small bioactive molecules: finding the needle in the haystack. Angew Chem Int Ed 2013; 52: 2744–92. 10.1002/anie.201208749 23418026

[34] Chen KG , Mallon BS, Park K et al. Pluripotent stem cell platforms for drug discovery. Trends Mol Med 2018; 24: 805–20. 30006147 10.1016/j.molmed.2018.06.009 PMC6117164

[35] Li J , Xu Y, Long XD et al. Cbx4 governs HIF-1 alpha to potentiate angiogenesis of hepatocellular carcinoma by its SUMO E3 ligase activity. Cancer Cell 2014; 25: 118–31. 24434214 10.1016/j.ccr.2013.12.008

[36] Huang Y , Lin D, Taniguchi CM. Hypoxia inducible factor (HIF) in the tumor microenvironment: friend or foe? Sci China Life Sci 2017; 60: 1114–24. 29039125 10.1007/s11427-017-9178-y PMC6131113

[37] Bhattarai D , Xu X, Lee K. Hypoxia-inducible factor-1 (HIF-1) inhibitors from the last decade (2007 to 2016): a ‘structure-activity relationship’ perspective. Med Res Rev 2018; 38: 1404–42. 10.1002/med.21477 29278273

[38] Xia XJ , Zuo FF, Luo MG et al. Role of TRIM33 in Wnt signaling during mesendoderm differentiation. Sci China Life Sci 2017; 60: 1142–9. 28844090 10.1007/s11427-017-9129-3

[39] Qu Y , Olsen JR, Yuan X et al. Small molecule promotes beta-catenin citrullination and inhibits Wnt signaling in cancer. Nat Chem Biol 2018; 14: 94–101. 29083417 10.1038/nchembio.2510

[40] Wang W , Liu HY, Wang S et al. A diterpenoid derivative 15-oxospiramilactone inhibits Wnt/beta-catenin signaling and colon cancer cell tumorigenesis. Cell Res 2011; 21: 730–40. 21321609 10.1038/cr.2011.30 PMC3203668

[41] Wang S , Yin JL, Chen DZ et al. Small-molecule modulation of Wnt signaling via modulating the Axin-LRP5/6 interaction. Nat Chem Biol 2013; 9: 579–85. 23892894 10.1038/nchembio.1309

[42] Willoughby LF , Schlosser T, Manning SA et al. An in vivo large-scale chemical screening platform using *Drosophila* for anti-cancer drug discovery. Dis Models Mech 2013; 6: 521–9. 10.1242/dmm.009985 PMC3597034 22996645

[43] Ridges S , Heaton WL, Joshi D et al. Zebrafish screen identifies novel compound with selective toxicity against leukemia. Blood 2012; 119: 5621–31. 22490804 10.1182/blood-2011-12-398818 PMC3382926

[44] Kalin RE , Banziger-Tobler NE, Detmar M et al. An in vivo chemical library screen in Xenopus tadpoles reveals novel pathways involved in angiogenesis and lymphangiogenesis. Blood 2009; 114: 1110–22. 19478043 10.1182/blood-2009-03-211771 PMC2721788

[45] Futamura Y , Muroi M, Osada H. Target identification of small molecules based on chemical biology approaches. Mol Biosyst 2013; 9: 897–914. 23354001 10.1039/c2mb25468a

[46] Ziegler S , Pries V, Hedberg C et al. Target identification for small bioactive molecules: finding the needle in the haystack. Angew Chem Int Ed 2013; 52: 2744–92. 10.1002/anie.201208749 23418026

[47] Ito T , Ando H, Suzuki T et al. Identification of a primary target of thalidomide teratogenicity. Science 2010; 327: 1345–50. 20223979 10.1126/science.1177319

[48] Roemer T , Davies J, Giaever G et al. Bugs, drugs and chemical genomics. Nat Chem Biol 2012; 8: 46–56. 10.1038/nchembio.744 22173359

[49] Schirle M , Bantscheff M, Kuster B. Mass spectrometry-based proteomics in preclinical drug discovery. Chem Biol 2012; 19: 72–84. 22284356 10.1016/j.chembiol.2012.01.002

[50] Berrade L , Garcia AE, Camarero JA. Protein microarrays: novel developments and applications. Pharm Res 2011; 28: 1480–99. 21116694 10.1007/s11095-010-0325-1 PMC3137928

[51] Tao SC , Chen CS, Zhu H. Applications of protein microarray technology. Comb Chem High Throughput Screen 2007; 10: 706–18. 18045082 10.2174/138620707782507386

[52] Huang J , Zhu H, Haggarty SJ et al. Finding new components of the target of rapamycin (TOR) signaling network through chemical genetics and proteome chips. Proc Natl Acad Sci USA 2004; 101: 16594–9. 15539461 10.1073/pnas.0407117101 PMC527135

[53] Bae N , Viviano M, Su XN et al. Developing Spindlin1 small-molecule inhibitors by using protein microarrays. Nat Chem Biol 2017; 13: 750–6. 28504676 10.1038/nchembio.2377 PMC5831360

[54] Henzler-Wildman K , Kern D. Dynamic personalities of proteins. Nature 2007; 450: 964–72. 18075575 10.1038/nature06522

[55] Xu Y , Liu J, Wu Y et al. Natural products against hematological malignancies and identification of their targets. Sci China Life Sci 2015; 58: 1191–201. 26566803 10.1007/s11427-015-4922-4

[56] Zhen T , Wu CF, Liu P et al. Targeting of AML1-ETO in t(8;21) leukemia by oridonin generates a tumor suppressor-like protein. Sci Transl Med 2012; 4: 127ra38. 10.1126/scitranslmed.3003562 22461642

[57] Wang X , Lin Q, Lv F et al. LG-362B targets PML-RAR alpha and blocks ATRA resistance of acute promyelocytic leukemia. Leukemia 2016; 30: 1465–74. 27012866 10.1038/leu.2016.50

[58] Gu ZM , Wu YL, Zhou MY et al. Pharicin B stabilizes retinoic acid receptor-alpha and presents synergistic differentiation induction with ATRA in myeloid leukemic cells. Blood 2010; 116: 5289–97. 20739655 10.1182/blood-2010-02-267963

[59] Xu HZ , Huang Y, Wu YL et al. Pharicin A, a novel natural ent-kaurene diterpenoid, induces mitotic arrest and mitotic catastrophe of cancer cells by interfering with BubR1 function. Cell Cycle 2010; 9: 2969–79. 10.4161/cc.9.14.12406 PMC3233523 20603598

[60] Zhang RH , Liu ZK, Yang DS et al. Phytochemistry and pharmacology of the genus Leonurus: the herb to benefit the mothers and more. Phytochemistry 2018; 147: 167–83. 29335190 10.1016/j.phytochem.2017.12.016

[61] Liu CX , Yin QQ, Zhou HC et al. Adenanthin targets peroxiredoxin I and II to induce differentiation of leukemic cells. Nat Chem Biol 2012; 8: 486–93. 22484541 10.1038/nchembio.935

[62] Liu CX , Zhou HC, Yin QQ et al. Targeting peroxiredoxins against leukemia. Exp Cell Res 2013; 319: 170–6. 22728267 10.1016/j.yexcr.2012.06.013

[63] Schieber M , Chandel NS. ROS function in redox signaling and oxidative stress. Curr Biol 2014; 24: R453–62. 24845678 10.1016/j.cub.2014.03.034 PMC4055301

[64] Kang SW , Lee S, Lee EK. ROS and energy metabolism in cancer cells: alliance for fast growth. Arch Pharm Res 2015; 38: 338–45. 25599615 10.1007/s12272-015-0550-6

[65] Yue Z , Xiao X, Wu J et al. *ent*-Jungermannenone C triggers reactive oxygen species-dependent cell differentiation in leukemia cells. J Nat Prod 2018; 81: 298–306. 29394050 10.1021/acs.jnatprod.7b00722

[66] Ying M , Shao X, Jing H et al. Ubiquitin-dependent degradation of CDK2 drives the therapeutic differentiation of AML by targeting PRDX2. Blood 2018; 131: 2698–711. 29720484 10.1182/blood-2017-10-813139

[67] Rossari F , Minutolo F, Orciuolo E. Past, present, and future of Bcr-Abl inhibitors: from chemical development to clinical efficacy. J Hematol Oncol 2018; 11: 84. 29925402 10.1186/s13045-018-0624-2 PMC6011351

[68] Liu C , Nie D, Li J et al. Antitumor effects of blocking protein neddylation in T315I-BCR-ABL leukemia cells and leukemia stem cells. Cancer Res 2018; 78: 1522–36. 29321163 10.1158/0008-5472.CAN-17-1733

[69] Dong B , Liang Z, Chen Z et al. Cryptotanshinone suppresses key onco-proliferative and drug-resistant pathways of chronic myeloid leukemia by targeting STAT5 and STAT3 phosphorylation. Sci China Life Sci 2018; 61: 999–1009. 30054832 10.1007/s11427-018-9324-y

[70] Sun W , Xie Z, Liu Y et al. JX06 selectively inhibits pyruvate dehydrogenase kinase PDK1 by a covalent cysteine modification. Cancer Res 2015; 75: 4923–36. 26483203 10.1158/0008-5472.CAN-15-1023

[71] Okoye-Okafor UC , Bartholdy B, Cartier J et al. New IDH1 mutant inhibitors for treatment of acute myeloid leukemia. Nat Chem Biol 2015; 11: 878–86. 26436839 10.1038/nchembio.1930 PMC5155016

[72] Chen B , Zang W, Wang J et al. The chemical biology of sirtuins. Chem Soc Rev 2015; 44: 5246–64. 25955411 10.1039/c4cs00373j

[73] Sebastian C , Zwaans BMM, Silberman DM et al. The histone deacetylase SIRT6 is a tumor suppressor that controls cancer metabolism. Cell 2012; 151: 1185–99. 23217706 10.1016/j.cell.2012.10.047 PMC3526953

[74] Kugel S , Sebastian C, Fitamant J et al. SIRT6 suppresses pancreatic cancer through control of Lin28b. Cell 2016; 165: 1401–15. 27180906 10.1016/j.cell.2016.04.033 PMC4892983

[75] Huang WK , Lu SY, Huang ZM et al. Allosite: a method for predicting allosteric sites. Bioinformatics 2013; 29: 2357–9. 23842804 10.1093/bioinformatics/btt399

[76] Huang ZM , Zhao ZX, Deng W et al. Identification of a cellularly active SIRT6 allosteric activator. Nat Chem Biol 2018; 14: 1118–26. 30374165 10.1038/s41589-018-0150-0

[77] Gao YH , Wu ZX, Xie LQ et al. VHL deficiency augments anthracycline sensitivity of clear cell renal cell carcinomas by down-regulating ALDH2. Nat Commun 2017; 8: 15337. 28643803 10.1038/ncomms15337 PMC5481740

[78] Guo ZQ , Zheng T, Chen B et al. Small-molecule targeting of E3 ligase adaptor SPOP in kidney cancer. Cancer Cell 2016; 30: 474–84. 27622336 10.1016/j.ccell.2016.08.003

[79] Li X , Wang W, Chen J. Recent progress in mass spectrometry proteomics for biomedical research. Sci China Life Sci 2017; 60: 1093–113. 29039124 10.1007/s11427-017-9175-2

[80] Macalino SJY , Basith S, Clavio NAB et al. Evolution of in silico strategies for protein-protein interaction drug discovery. Molecules 2018; 23: E1963. 30082644 10.3390/molecules23081963 PMC6222862

[81] Grembecka J , He S, Shi A et al. Menin-MLL inhibitors reverse oncogenic activity of MLL fusion proteins in leukemia. Nat Chem Biol 2012; 8: 277–84. 22286128 10.1038/nchembio.773 PMC3401603

[82] Illendula A , Pulikkan JA, Zong H et al. Chemical biology. A small-molecule inhibitor of the aberrant transcription factor CBFbeta-SMMHC delays leukemia in mice. Science 2015; 347: 779–84. 25678665 10.1126/science.aaa0314 PMC4423805

[83] Jiang H , Deng R, Yang X et al. Peptidomimetic inhibitors of APC-Asef interaction block colorectal cancer migration. Nat Chem Biol 2017; 13: 994–1001. 28759015 10.1038/nchembio.2442

[84] Jiang H , Hsieh-Wilson L, Arruda P et al. Voices of chemical biology. Nat Chem Biol 2015; 11: 446–7. 26083062 10.1038/nchembio.1845

[85] Zhu H , Tamura T, Hamachi I. Chemical proteomics for subcellular proteome analysis. Curr Opin Chem Biol 2019; 48: 1–7. 30170243 10.1016/j.cbpa.2018.08.001

[86] Li PP , Li JJ, Wang L et al. Proximity labeling of interacting proteins: application of BioID as a discovery tool. Proteomics 2017; doi: 10.1002/pmic.201700002. 10.1002/pmic.201700002 28271636

[87] Roux KJ , Kim DI, Burke B et al. BioID: a screen for protein-protein interactions. Curr Protoc Protein Sci 2018; 91: 19.23.1–15. 29516480 10.1002/cpps.51 PMC6028010

[88] Liu Q , Zheng J, Sun WP et al. A proximity-tagging system to identify membrane protein-protein interactions. Nat Methods 2018; 15: 715–22. 30104635 10.1038/s41592-018-0100-5

[89] Zhao Y , Jin J, Hu Q et al. Genetically encoded fluorescent sensors for intracellular NADH detection. Cell Metab 2011; 14: 555–66. 21982715 10.1016/j.cmet.2011.09.004 PMC3253140

[90] Zhao Y , Hu Q, Cheng F et al. SoNar, a highly responsive NAD^+^/NADH sensor, allows high-throughput metabolic screening of anti-tumor agents. Cell Metab 2015; 21: 777–89. 25955212 10.1016/j.cmet.2015.04.009 PMC4427571

[91] Tao R , Zhao Y, Chu H et al. Genetically encoded fluorescent sensors reveal dynamic regulation of NADPH metabolism. Nat Meth 2017; 14: 720–8. 10.1038/nmeth.4306 PMC5555402 28581494

[92] Wang X , Chen XJ, Yang Y. Spatiotemporal control of gene expression by a light-switchable transgene system. Nat Methods 2012; 9: 266–9. 22327833 10.1038/nmeth.1892

[93] Chen XJ , Li T, Wang X et al. Synthetic dual-input mammalian genetic circuits enable tunable and stringent transcription control by chemical and light. Nucleic Acids Res 2016; 44: 2677–90. 26673714 10.1093/nar/gkv1343 PMC4824083

[94] Chen XJ , Liu RM, Ma ZC et al. An extraordinary stringent and sensitive light-switchable gene expression system for bacterial cells. Cell Res 2016; 26: 854–7. 27311594 10.1038/cr.2016.74 PMC5129885

[95] Imayoshi I , Isomura A, Harima Y et al. Oscillatory control of factors determining multipotency and fate in mouse neural progenitors. Science 2013; 342: 1203–8. 24179156 10.1126/science.1242366

[96] Fan XY , Li J, Chen PR. Bioorthogonal chemistry in living animals. Natl Sci Rev 2017; 4: 300–2.

[97] Li J , Yu J, Zhao J et al. Palladium-triggered deprotection chemistry for protein activation in living cells. Nat Chem 2014; 6: 352–61. 24651204 10.1038/nchem.1887

[98] Li J , Jia S, Chen PR. Diels-Alder reaction-triggered bioorthogonal protein decaging in living cells. Nat Chem Biol 2014; 10: 1003–5. 25362360 10.1038/nchembio.1656

[99] Zhang G , Li J, Xie R et al. Bioorthogonal chemical activation of kinases in living systems. ACS Cent Sci 2016; 2: 325–31. 27280167 10.1021/acscentsci.6b00024 PMC4882735

[100] Zhong F , Xing J, Li X et al. Artificial intelligence in drug design. Sci China Life Sci 2018; 61: 1191–204. 30054833 10.1007/s11427-018-9342-2

[101] Zhang L , Tan J, Han D et al. From machine learning to deep learning: progress in machine intelligence for rational drug discovery. Drug Discov Today 2017; 22: 1680–5. 28881183 10.1016/j.drudis.2017.08.010

